# Smartphone measurements of physical activity and fitness are associated with early trial discontinuation of patients in (hemato)oncology phase I/II clinical trials

**DOI:** 10.1007/s00520-020-05902-2

**Published:** 2020-11-24

**Authors:** Joeri A. J. Douma, Sonja Zweegman, Mieke Alberts, Sandy Kruyswijk, Niels C. W. J. van de Donk, Myra van Linde, Laurien M. Buffart, Henk M. W. Verheul

**Affiliations:** 1 Department of Medical Oncology, Cancer Center Amsterdam, Amsterdam University Medical Centers, Vrije Universiteit Amsterdam, De Boelelaan 1117, Amsterdam, The Netherlands; 2 Department of Hematology, Cancer Center Amsterdam, Amsterdam University Medical Centers, Vrije Universiteit Amsterdam, De Boelelaan 1117, Amsterdam, The Netherlands; 3 Department of Physiology, Radboudumc, Geert Grooteplein 10, Nijmegen, The Netherlands; 4 Department of Medical Oncology, Radboudumc, Geert Grooteplein 10, Nijmegen, The Netherlands

**Keywords:** Smartphone measurements, Physical activity, Physical fitness, Early trial discontinuation

## Abstract

**Background:**

Patients, who discontinue early, do not benefit from phase I/II clinical trials (early-phase clinical trials (EPCT)). In this study, associations between objective smartphone measurements of physical activity and fitness and early trial discontinuation in patients with cancer participating in EPCT were investigated.

**Methods:**

Before start of treatment, physical activity (steps/day) and physical fitness (meters walked in 6 min) were measured with a smartphone, and patient-reported physical function (PRO-PF) was assessed (EORTC QLQ-C30-PF). Early trial discontinuation was defined as discontinuation ≤ 28 days. Univariable logistic regression analyses were performed to study associations of physical activity, fitness, and function with early trial discontinuation. Optimal cutoff values of physical activity and fitness were assessed with ROCs, based on positive predictive values (PPV).

**Results:**

Median (interquartile range (IQR)) step count was 4263 (2548–6897) steps/day, mean ± standard deviation 6-min walking distance was 477 ± 120 m and median (IQR) PRO-PF score was 83 (67–95) points. Fourteen patients (12%) discontinued the trial early. Smartphone measurements of physical activity in units of 100 steps per day (odds ratio (OR) = 0.96, 95% CI = 0.94–0.99, *p* = 0.01), physical fitness (OR = 0.99, 95% CI = 0.98–0.99, *p* < 0.01), and PRO-PF (OR = 0.97, 95% CI = 0.94–1.00, *p* = 0.03) were associated with early trial discontinuation. Optimal cutoff values were < 900 steps for physical activity and < 285 m for physical fitness. PPV for early trial discontinuation was 100% in patients who walked both < 1500 steps per day and < 300 m in 6 min.

**Conclusions:**

Objective smartphone measurements of physical activity and fitness are associated with early trial discontinuation. However, cutoff values should be externally validated in a larger cohort before implementation in clinical practice.

## Introduction

Patients with hematologic malignancies or solid tumors, who are refractory to standard therapies, are candidates to participate in early-phase (phase I or II) clinical trials (EPCT). Phase I clinical trials establish the maximal tolerated dosage of a possible effective anti-cancer drug [1]. This recommended dose can subsequently be used in phase II and III trials to investigate effectiveness of the drug [2]. To be eligible for participation in EPCT, patients must have an adequate performance status and organ function, and a minimal life expectancy of 3 months without treatment [2–4]. By applying these rigorous inclusion criteria, potential harm of study treatment could be minimized while supporting optimal evaluation of the novel treatment strategy.

Previous studies revealed that despite stringent selection, 15–20% of patients with cancer participating in phase I trials discontinued early [5–7] due to reasons other than dose-limiting toxicity (DLT). Specific rates of early trial discontinuation in phase II trials are unknown, but are likely lower than rates in phase I clinical trials. Patients who discontinue trials of experimental and mostly toxic therapies within a month do not benefit from study treatment, and participation of these patients in the final stage of their lives should be prevented. Approximately 70% of early trial discontinuation is due to a deteriorating physical function caused by progressive disease and/or concomitant medical events that are not related to study treatment [5]. Drug development is delayed by patients who discontinue trial participation during the evaluation period because of non-drug-related events. These patients need to be replaced for evaluation of safety and DLT and thereby hamper optimal conduct of EPCT [5, 8].

To minimize early trial discontinuation in patients participating in EPCT, it is important to adequately identify them upfront. Patients eligible for EPCT have already exhausted all or at least several available effective therapies under standard care. Although organ functions might be preserved, patients often have impaired physical function as a consequence of previous treatments or progression of the malignancy. Objective measurements of physical activity and fitness might provide a more accurate estimation of a patient’s physical function than the subjective and rough Eastern Cooperative Oncology Group/World Health Organization (ECOG/WHO) performance score (PS) that is often used in clinical practice [9–11]. Smartphone measurements of physical activity and fitness might overcome problems that are experienced with commonly used objective measurements (e.g., accelerometer, six-minute walk test (6MWT) inside hospital) [12], such as high time investment and costs, because nowadays, most patients with cancer already possess a smartphone [13, 14]. In a previous study, we found that objective smartphone measurements of step count are feasible, valid, and reliable in patients with cancer [15]. Furthermore, smartphone measurements of physical fitness based on the Global Positioning System (GPS) are accurate, valid, and reliable [15, 16].

The added value of smartphone measurements of physical activity and fitness in clinical oncology practice is currently unknown. The primary aim of this study was to determine whether objective smartphone measurements of physical activity and fitness are associated with early trial discontinuation in patients participating in EPCT. Acceptability, feasibility, and usability of smartphone measurements of physical activity and fitness in clinical practice were also evaluated.

## Methods

### Study design

This is an observational study for patients with advanced cancer referred for participation in EPCT to determine whether objective smartphone measurements of physical activity and fitness are associated with early trial discontinuation. Patients were recruited from the outpatient Medical Oncology and Hematology department of Amsterdam University Medical Centers (Amsterdam UMC), location VUmc. All patients with a hematological malignancy or solid tumor who were planned to participate in a phase I or II clinical trial were eligible for this study. Exclusion criteria were (i) insufficient mastery of Dutch language, (ii) presence of cognitive disorders or severe emotional instability, and (iii) physical impairments limiting walking ability (e.g., bone fracture or paraplegia).

### Outcome measures

Early trial discontinuation was defined as trial discontinuation within 28 days after start in EPCT. Acceptability of smartphone measurements in clinical practice was defined as the proportion of eligible patients who were actually included in the study. Feasibility was measured as the proportion of valid measurements of physical activity, fitness, and PRO-PF. Usability was assessed with the system usability scale (SUS), a 10-item questionnaire designed and validated to assess usability of electronic systems [17], for which a score ≥ 70 is considered good usability [17].

### Measurements of physical activity, fitness, and function

Patients were instructed to wear the smartphone (IPhone SE, iOS 10.2) for 7 consecutive days in the hip-waist region either in their pocket or attached to a belt, during all waking hours. Physical activity was defined as mean number of steps per day (step count). To calculate the mean daily number of steps per day, at least 3 valid days of wear-time were needed [18]. Due to the inability to perform a detailed analysis of wear-time for smartphone measurements, every day that the smartphone had recorded any steps was considered a valid wear day [15].

Physical fitness was assessed via the distance that a person can walk in 6 min (6MWT) [19]. Patients were instructed to perform a 6WMT outdoors in their home environment using a smartphone application (Walkmeter), which used the GPS signal to assess traveled distance. Patients were instructed to choose an outdoor environment which allowed walking in an almost straight line without any need to stop during the test. Data of patients who encountered significant technical problems during the 6WMT or who had not performed the 6MWT properly were regarded as missing values.

Patient-reported physical function (PRO-PF) was measured using the EORTC QLQ-C30 physical functioning subscale, which consists of 5 questions. A score below 66.7 was considered poor physical function [20].

### Covariables

Age, gender, primary tumor type, performance score, height, weight, opioids use, number of metastatic sites, and comorbidity level were retrieved from the medical records. BMI was calculated based on the objective measurements of height and weight (body weight/height^2^, kg/m^2^). The comorbidity level was assessed with the Charlson Comorbidity Index [21].

### Statistical analysis

Based on an expected early trial discontinuation rate of 20% in the first 3 weeks in phase I trials [5–7], a sample size of 135 patients was required in order to select 4 independent predictors with sufficient power (based on the rule of thumb of 1 predictor per 10 events) [22]. Acceptability, feasibility, and usability of smartphone measurements of physical activity, physical fitness, and PRO-PF were described as mean ± standard deviation (SD) in case of normal distributions or median (interquartile range (IQR)) otherwise. A chi-square test was used to compare the rate of early trial discontinuation in patients with a PS of 0 to patients with a PS > 0 and to compare the rate of early trial discontinuation in patients with a valid 6MWT to patients without a valid 6MWT. An independent samples *t* test was used to compare the mean score of usability in patients with a valid 6MWT to patients without a valid 6MWT.

Univariable logistic regression analyses were performed to assess whether physical activity, physical fitness, and PRO-PF were associated with early trial discontinuation. Odds ratios, with 95% confidence intervals and level of significance, are presented. In order to facilitate interpretability for physical activity, odds ratio and 95% confidence interval are presented for every increase in 100 steps per day. Furthermore, area under the receiver operator curve (ROC) was calculated to determine which cutoff value of physical activity and physical fitness best predicted early trial discontinuation. With information on sensitivity and specificity derived from the ROC curve, we calculated the positive predictive value (PPV) of cutoff values using Bayes theorem PPV formula: PPV = sensitivity × prevalence/((sensitivity × prevalence) + ((1 − prevalence) × (1 − specificity)). In addition, the combination of cutoff values of physical activity and fitness were evaluated. The cutoff values with the highest PPV with corresponding sensitivity, specificity, positive predictive value, and number needed to screen (NNS) were reported. The number needed to screen was defined as the number of patients who needed to be measured in order to identify one patient with early trial discontinuation. A sensitivity analysis was performed to calculate the PPV and NNS in a subgroup of patients with PS > 0. *p* < 0.05 was considered statistically significant in all analyses.

## Results

### Patient recruitment and characteristics

**Fig. 1:**
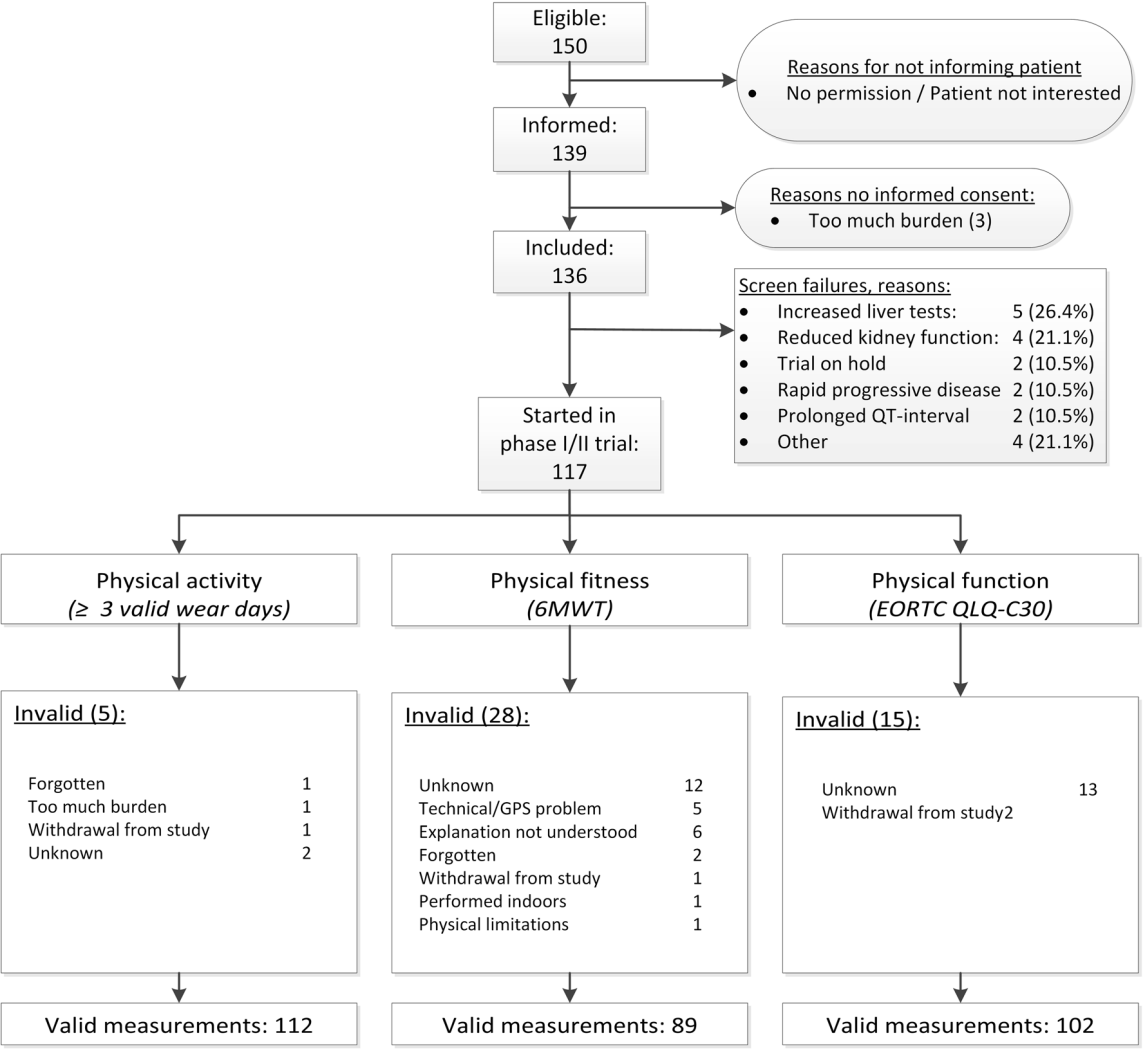
Valid measurements of physical activity, physical fitness, and physical function. 6MWT = six-minute walk test, EORTC = European Organisation for Research and Treatment for Cancer, QLQ-C30 = Quality of Life Questionnaire-C30, GPS = global positioning system

Between October 2017 and December 2019, 136 of the 150 eligible patients were included in this study, of whom 117 started systemic treatment in the EPCT (Fig. 1). The main reasons for the 19 screen failures were liver test (26%) and kidney function (22%) abnormalities that did not meet the eligibility criteria of the EPCT (Fig. 1). The mean age of the 117 patients who started treatment was 63 ± 11 years, 42% were women and most common primary tumor types were gastrointestinal cancer (27%), multiple myeloma (17%), and glioblastoma (13%) (Table 1). Fourteen (12%) of the 117 patients discontinued the trial prematurely and 18 (16%) patients died within 90 days from the start of treatment in the EPCT (Table 2). Main reasons for early trial discontinuation were progressive disease (36%), toxicity (29%), and insufficient physical condition of the patient (14%) (Table 2). The rate of early trial discontinuation was significantly different with respect to performance status, 4% in patients with a PS of 0 and 18% in patients with a PS > 0 (*p* = 0.02).

**Table 1:** Baseline characteristics of patients (*n* = 117)

Age, mean, years	63 ± 11
Sex, *n* (%) women	49 (42)
BMI (kg/m^2^), mean	26.7 ± 5.8
Use of opioids, *n* (%)	25 (21)
Primary tumor, *n* (%)	
Gastrointestinal	32 (27)
Multiple myeloma	20 (17)
Glioblastoma	15 (13)
Breast cancer	10 (9)
Lymphoma	8 (7)
Head and neck cancer	7 (6)
Lung cancer	6 (5)
Genitourinary	5 (4)
Waldenström’s macroglobulinemia	3 (3)
Chronic lymphatic leukemia (CLL)	3 (3)
Gynecologic cancers	3 (3)
Others	
Neuroendocrine carcinoma	2 (2)
Thyroid cancer	1 (1)
Sarcoma	1 (1)
Myoepithelial carcinoma	1 (1)
ECOG/WHO performance status, *n* (%)^a^	
0	49 (42)
1	63 (54)
2	4 (3)
Number of metastatic sites^b^ (*n*)	2.3 ± 1.7
Charlson comorbidity elevated (primary malignancy excluded), *n* (%)	31 (27)
Diabetes mellitus (*n*)	14
COPD (*n*)	12
Cerebrovascular accident or transient ischemic attack (*n*)	5
Myocardial infarction (*n*)	3
Peripheral vascular disease (*n*)	1
Malignant lymphoma (*n*)	1
Peptic ulcer disease (*n*)	1
Physical activity, step count per day, median (IQR)^c^	4263 (2548–6897)
Physical fitness, distance in meters walked in 6 min, mean^d^	477 ± 120
Physical function, EORTC QLQ-C30 physical function subscale score, median (IQR)^e^	83 (67–95)
Usability, system usability scale score, median (IQR)^f^	80 (58–90)

^a^*n—*1

^b^*n*—34

^c^*n*—5

^d^− 28

^e^− 15

^f^− 25

*SD* standard deviation, *BMI* body mass index, *ECOG/WHO* Eastern Cooperative Oncology Group/World Health Organization, *COPD* chronic obstructive pulmonary disease, *EORTC* European Organisation for Research and Treatment for Cancer, *QLQ-C30* Quality of Life Questionnaire-C30, *IQR* interquartile range

**Table 2:** Reasons for early trial discontinuation

Early trial discontinuation, *n* (%)	14 (12)
Reasons early trial discontinuation, *n* (%)	
Progressive disease	5 (36)
Toxicity	4 (29)
Patient’s condition	2 (14)
Patient’s request	1 (8)
Comorbidity/complication of previous treatment	1 (8)
Death	1 (8)
90-day mortality, *n* (%)^a^	18 (16)

^a^*n*—2

### Acceptability, feasibility, and usability

As described above, 136 of the 150 eligible patients were included in this study, resulting in an acceptability rate of 91% (Fig. 1). Regarding feasibility, valid measurements of physical activity, physical fitness, and physical function were available for 96%, 76%, and 87% of the 117 patients who started treatment in the EPCT (Fig. 1). The proportion of patients with early trial discontinuation was 18% in patients without a valid 6MWT and 10% in patients with a valid 6MWT (*p* = 0.28). The median (IQR) usability score was 80 (58–90) (Table 1). Patients without a valid 6MWT had a significantly 10 points lower usability score than patients with a valid 6MWT (*p* = 0.02).

### Physical activity and association with early trial discontinuation

**Fig. 2:**
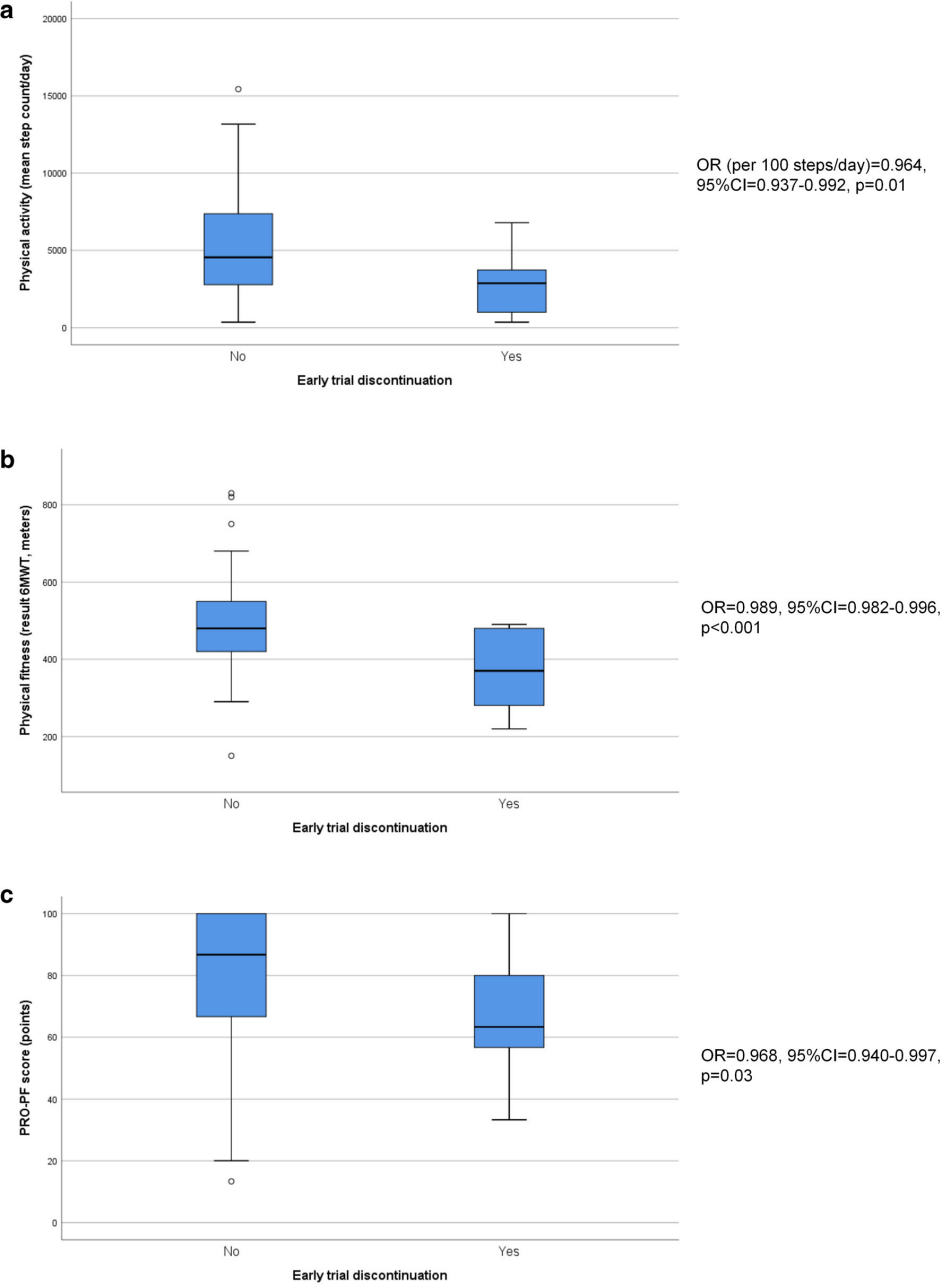
Graphic representation of the association between physical activity (**a**), physical fitness (**b**) and PRO-PF (**c**) and early trial discontinuation. 6MWT = six-minute walk test, PRO-PF = patient-reported outcome of physical function, OR = odds ratio, 95% CI = 95% confidence interval

The median (IQR) step count was 4263 (2548–6897) steps per day, ranging from a minimum of 342 to a maximum 15,444 steps per day. Lower physical activity (per 100 steps per day: OR = 0.964, 95% CI = 0.937–0.992) was significantly associated with early trial discontinuation (Fig. 2). The optimal cutoff value was 900 steps for physical activity. Three of the 6 patients (PPV = 50%) with a step count < 900 steps per day discontinued the trial early (Table 3).

**Table 3:** Positive predictive value, sensitivity, specificity, and number needed to screen of best cutoff values of physical activity, fitness, and PRO-PF

	Number of patients	Early trial discontinuation	No early trial discontinuation	PPV (%)	Sensitivity (%)	Specificity (%)	Number needed to screen
Physical activity							
< 900 steps	6/112	3	3	50	21	97	37
Physical fitness							
< 285 m	4/89	3	1	75	33	99	30
PRO-PF							
< 66.7	23/102	6	17	26	50	81	17
Physical activity < 1500 steps and fitness < 300 m	3/89	3	0	100	33	100	30
Subgroup of patients with PS > 0							
Physical activity < 900 steps	5/65	3	2	60	25	98	21
Physical fitness < 285 m	3/48	2	1	67	29	98	24
Physical activity < 1500 steps and fitness < 300 m	3/48	3	0	100	43	100	16

*PRO-PF* patient-reported physical function, *PPV* positive predictive value, *PS* performance status

### Physical fitness and association with early trial discontinuation

The mean distance walked during the 6MWT was 477 ± 120 m, with a range from 150 to 830 m. Lower physical fitness (per meter walked: OR = 0.989, 95% CI = 0.982–0.996) was significantly associated with early trial discontinuation and the optimal cutoff value was 285 m (Fig. 2). Three of the four patients (PPV = 75%) who walked less than 285 m during the 6MWT discontinued the trial early (Table 3).

### PRO-PF and association with early trial discontinuation

The median (IQR) PRO-PF score was 83 (67–95) points, with a range from 13 to 100 points. Lower PRO-PF (OR = 0.968, 95% CI = 0.940–0.997) was significantly associated with early trial discontinuation (Fig. 2). Seven of the 35 patients (PPV = 20%) with a poor PRO-PF discontinued the trial early.

### Combined analysis in association with early trial discontinuation

The best cutoff values for the combination of physical activity and physical fitness were < 1500 steps per day and < 300 m walked during the 6MWT. All three patients (PPV = 100%) with a step count < 1500 and a 6MWT < 300 m discontinued the trial early (Table 3). The sensitivity analyses in patients with PS > 0 showed a slightly higher PPV for step counts < 900 but slightly lower PPV for a 6MWT < 285 m. However, the NNS was substantially lower (Table 3).

## Discussion

To the best of our knowledge, this is the first clinical study which shows that smartphone measurements of physical activity, physical fitness, and PRO-PF are significantly associated with early trial discontinuation in patients with cancer participating in EPCT. In order to identify patients for whom trial participation would not be feasible, we determined that the combination of a step count < 1500 steps per day and a distance < 300 m walked during the 6MWT identified these patients with a positive predictive value of 100%. This would indicate that in future EPCT, the inclusion of such patients should be discouraged.

The step count (median 4263, mean 4844) in this study is comparable with the mean step count of 4877 and 4800 steps per day in patients with advanced stage non-small cell lung cancer [23, 24] and a step count of 4057 per day found in patients with advanced cancer [15]. The mean distance of 477 m during the 6MWT in this study was comparable with the 425 m found in patients with advanced gastrointestinal and lung cancer [25] and the 424 m found in patients with advanced cancer [15]. The rate of early trial discontinuation of 12% was lower than observed in earlier studies [5, 6], which might be due to the inclusion of phase II trials in general and phase I trials with potentially less toxic drugs like targeted therapy and immunotherapy in this study.

Usability and user-friendliness of smartphone measurements was considered to be good in this study, which was in line with two earlier studies of smartphone measurements in patients with cancer [15, 26]. This study shows that even the use of smartphone measurements in clinical practice and not only in feasibility studies is still associated with high usability and user-friendliness. The 93% participation rate in this study reflects the high willingness and acceptability to perform smartphone measurements. This enthusiasm among patients is promising for further research and implementation of smartphone measurements in clinical practice and seems to be consistent with an earlier study, which found that 82% of the patients were willing to use a smartphone (application) if this was recommended by their physician [27]. The feasibility of smartphone measurements was very high for physical activity, which was in line with earlier studies [15, 28, 29]. On the contrary, the 76% valid measurement of physical fitness assessments with the smartphone indicates room for improvement. The significantly lower usability score in patients without a valid 6MWT might imply that feasibility could be increased by improving user-friendliness of the application and that measurements of physical fitness with a customized application based on recently identified factors of smartphone application adherence [30] might still be promising for use in clinical practice.

Although a multivariable logistic regression analysis to build a prediction model for early trial discontinuation with physical activity, fitness, PRO-PF, and PS as variables was not possible due to the lower than expected rate of early trial discontinuation, physical activity, physical fitness, and PRO-PF were all associated with early trial discontinuation in patients with cancer participating in EPCT. The associations of physical activity and fitness with early trial discontinuation have not been studied before, but earlier studies have shown that physical activity and fitness are predictive for hospital admissions [31] and mortality [31–36] in patients with cancer. The results of this study show that smartphone measurements of physical activity and fitness were superior to PRO-PF regarding the positive predictive value, which favors the use of smartphone measurements for patient selection in clinical practice. In particular, the 100% PPV of the combination of physical activity and fitness, with cutoff values of 1500 steps and 300 m, respectively, indicates high potential for use in clinical practice. Only three of the 14 patients (21%) with early trial discontinuation could be identified, which might be improved by a reduction in the number of invalid 6MWT. However, the improvement might be limited, as not all causes of early trial discontinuation will be predictable. On the other hand, a 20% reduction in early trial discontinuation can have a major impact on the quality of life of many patients as well as clinical trial efficiency worldwide. To reduce the number of patients needed to test, patients with a PS of 0 might be excluded from smartphone measurements of physical activity and fitness, because the rate of early trial discontinuation in these patients was very low. Based on these findings, the addition of smartphone measurements of physical activity and fitness to the eligibility criteria of EPCT is worth the consideration. However, the cutoff values should be externally validated prior to its use.

A possible limitation of this study and of future implementation in clinical practice might be that patients could get stimulated to be more physically active by wearing a device that measures their physical activity, although this was not the case in a study among healthy people [37]. Even if patients become more physically active by this mechanism, it might mean that their level of physical function is better than the patients who are unable to improve their level of physical activity, thus the selection may still be adequate. Another limitation of this study might be the relatively high number of invalid measurements for the 6MWT, which might be reduced in the future by improving the user-friendliness of the application and by sending reminders with extra information. Nevertheless, the association found between smartphone measurements of physical activity and fitness and early trial discontinuation shows high potential of these measurements, which supports further investigations.

Adjustments in the patient selection process of EPCT, to only allow fitter and more physically active patients, should be done cautiously because they potentially have far-reaching effects [38, 39]. Unnecessary restrictive eligibility criteria should be avoided to maintain generalizability to the patients who will ultimately be treated with the investigated drugs [40]. Eligibility criteria should only be constricted further if research indicates a risk for an adverse outcome, such as early trial discontinuation. When smartphone measurements are being used in clinical practice, the privacy of patients is extremely important and should be respected. Fortunately, the developers of smartphone applications have increasing experience with privacy issues in patients, which offer the opportunity to create a smartphone application that complies with all legal privacy regulations. The results of this study suggest that a more accurate estimation of physical function, by smartphone measurements of physical activity and fitness, could be of added value in the selection of patients for EPCT.

In conclusion, smartphone measurements of physical activity and fitness are associated with early trial discontinuation in patients with cancer participating in EPCT. These smartphone measurements of physical activity and fitness might be limited to patients with a suboptimal performance status in order to reduce the number of patients who need to perform measurements. The results indicate that these smartphone measures may have positive impact on trial conduct when added to the standard screening procedures. The cutoff values should however be externally validated in a larger cohort before implementation in clinical practice.
